# Supplementary material for: Equilibrium selection via current sheet relaxation and guide field amplification
Source: Nat Commun. 2023 Jan 10;14:139. doi: 10.1038/s41467-023-35821-9 (PMC9832116; doi:10.1038/s41467-023-35821-9)
Supplement: Supplementary file 1 — Supplementary Figures [file 41467_2023_35821_MOESM1_ESM.pdf]

Supplementary Materials

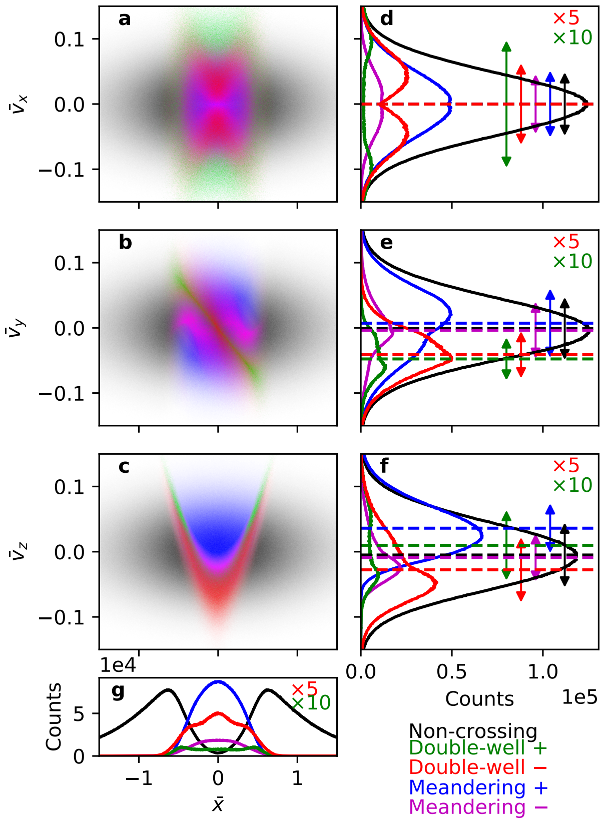

Figure S1: **Particle distribution in phase space, velocity space, and physical space.** Phase space distributions of the five orbit classes distinguished by the blue, magenta, green, red, black colors in **a** $(\bar{x}, \bar{v}_x)$, **b** $(\bar{x}, \bar{v}_y)$, and **c** $(\bar{x}, \bar{v}_z)$ spaces. Histograms of particles satisfying $\bar{x} > 0$ in **d** $\bar{v}_x$, **e** $\bar{v}_y$, and **f** $\bar{v}_z$ spaces. The dotted lines and arrows are respectively the average velocities and two standard deviations of each distribution. **g** Particle histogram in $\bar{x}$. The red and green lines in **d-g** are amplified five and ten times, respectively, so that they are visible on the same plot.

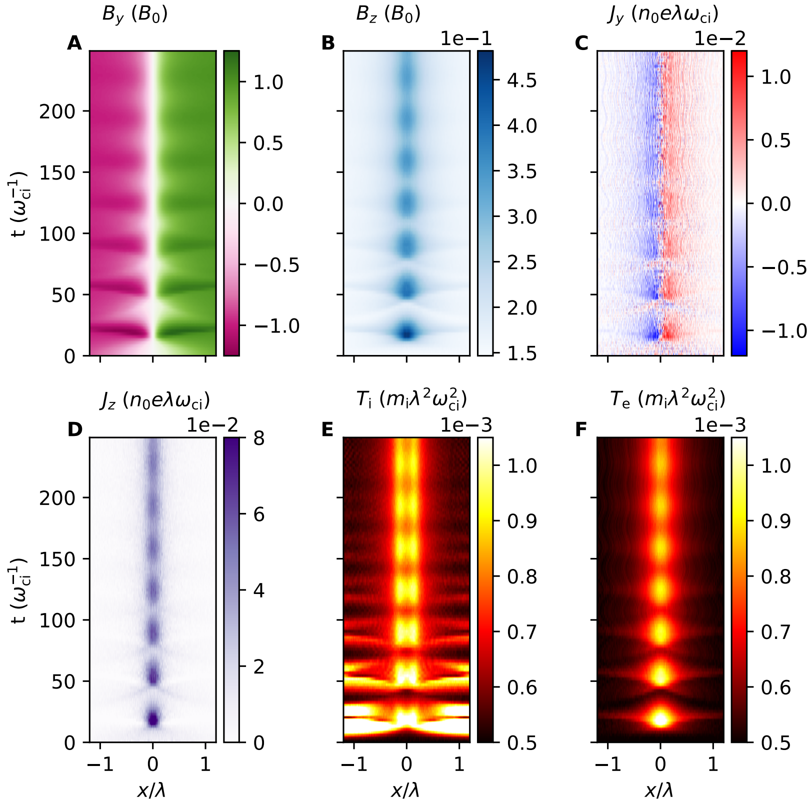

Figure S2: **Streak plots of variables from the particle-in-cell simulation with $c/v_A = 20$.** Same as Fig. 4, but for $c/v_A = 20$.

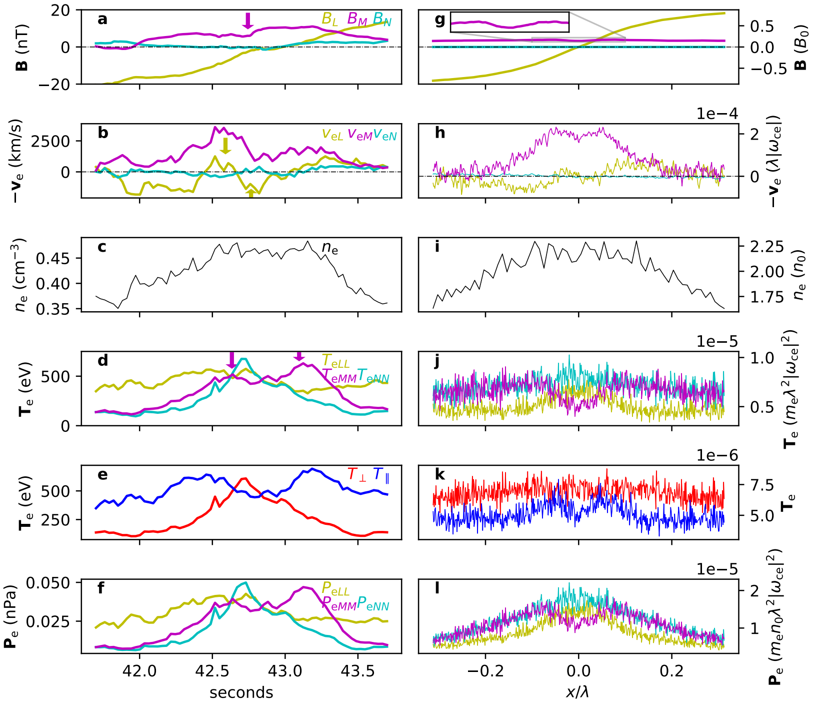

Figure S3: **Comparison of a simulated sheet with an initial dip in the guide field with MMS spacecraft observations.** Same as Fig. 7, but the simulated current sheet in the right panels had an initial dip in the guide field that carries over to the final configuration (inset in **g**).

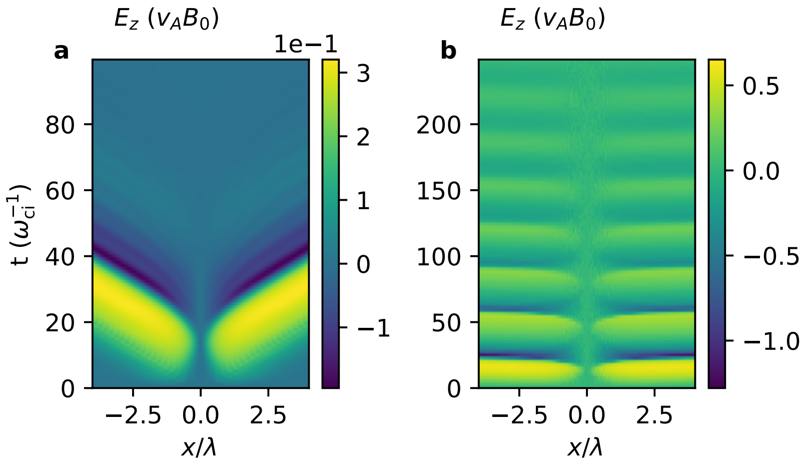

Figure S4: **Streak plots of E_z.** Streak plots of the inductive out-of-plane electric field E_z, for **a** $c/v_A = 2$ and **b** $c/v_A = 20$.

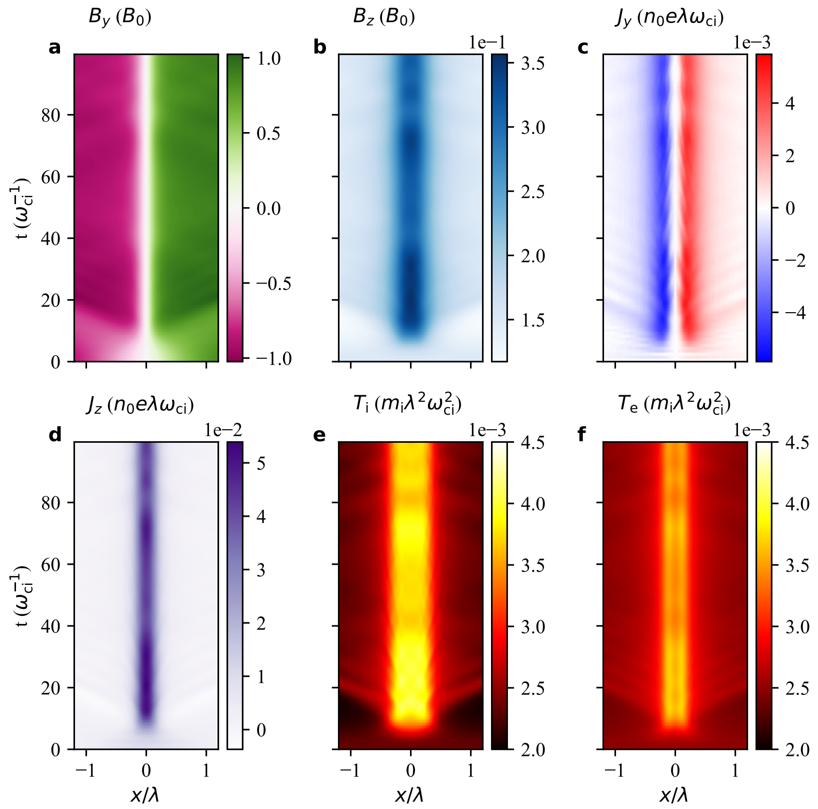

Figure S5: **Streak plots of variables from the particle-in-cell simulation with a low density.** Same as Fig. 4, but with an under-dense initial sheet with a temperature $T = T_{\text{eq}}$ and density $n = 0.2n_0$.
