# Supplementary material for: Equilibrium selection via current sheet relaxation and guide field amplification
Source: Nat Commun. 2023 Jan 10;14:139. doi: 10.1038/s41467-023-35821-9 (PMC9832116; doi:10.1038/s41467-023-35821-9)
Supplement: Supplementary file 3 — Editorial Assessment Report [file 41467_2023_35821_MOESM3_ESM.pdf]

nature portfolio

GUIDED OPEN ACCESS

nature physics | nature communications | communications physics

Contents of this report

1. **Manuscript details**: overview of your manuscript and the editorial team.
2. **Review synthesis**: summary of the reviewer reports provided by the editors.
3. **Editorial evaluations**: personalized evaluation and recommendation from all 3 journals.
4. **Annotated reviewer comments**: the referee reports with comments from the editors.
5. **Open research evaluation**: advice for adhering to best reproducibility practices.

About the editorial process

Because you selected the **Nature Portfolio Guided Open Access** option, your manuscript was assessed for suitability in three of our titles publishing high-quality work across the spectrum of physics research: ***Nature Physics***, ***Nature Communications***, and ***Communications Physics***. More information about Guided Open Access can be found here.

Collaborative editorial assessment

Your editorial team discussed the manuscript to determine its suitability for the Nature Portfolio Guided OA pilot. Our assessment of your manuscript takes into account several factors, including whether the work meets the **technical standard** of the Nature Portfolio and whether the findings are of **immediate significance** to the readership of at least one of the participating journals in the Nature Portfolio Guided Open Access physics cluster.

Peer review

Experts were asked to evaluate the following aspects of your manuscript:

- **Novelty** in comparison to prior publications;
- **Likely audience** of researchers in terms of broad fields of study and size;
- **Potential impact** of the study on the immediate or wider research field;
- **Evidence** for the claims and whether additional experiments or analyses could feasibly strengthen the evidence;
- **Methodological detail** and whether the manuscript is reproducible as written;
- Appropriateness of the **literature review**.

Editorial evaluation of reviews

Your editorial team discussed the potential suitability of your manuscript for each of the participating journals. They then discussed the revisions necessary in order for the work to be published, keeping each journal's specific editorial criteria in mind.

Journals in the Nature portfolio will support authors wishing to transfer their reviews and (where reviewers agree) the reviewers' identities to journals outside of Springer Nature.
If you have any questions about review portability, please contact our editorial office at guidedoa@nature.com.

nature portfolio

GUIDED OPEN ACCESS
nature physics | nature communications | communications physics

Manuscript details

Tracking number	Submission date	Decision date	Peer review type
GUIDEDOA-22-00435	Mar 6, 2022	Jul 8, 2022	Single-blind

Manuscript title	Author details
Equilibrium selection via current sheet relaxation and guide field amplification<br><br>**Preprint:** link if available	Young Dae Yoon<br><br>**Affiliation:** Pohang Accelerator Laboratory

Editorial assessment team

**Primary editor**	**Bart Verberck**<br>Home journal: ***Nature Physics***<br>ORCID: **0000-0002-9601-8840**<br>Email: **bart.verberck@nature.com**
**Other editors consulted**	**Andrea Taroni**<br>Home journal: ***Nature Physics***<br>ORCID: **0000-0001-9550-5754**
**About your primary editor**	After 10 years in academia, Bart originally joined Nature Physics in 2013, where he handled topics from condensed-matter physics and plasma physics. He also launched a column called 'Measure for Measure' on aspects of metrology. In July 2017, Bart moved to Berlin to become Regional Executive Editor for the newly established Nature Portfolio office in Germany, which he helped develop as an editorial and publishing hub with a focus on local outreach. Since 2021, he combines that capacity with the role of Senior Consulting Editor at Nature Physics, where he covers soft-condensed-matter physics and has a consulting role for Nature Communications.

Editorial assessment and review synthesis

**Editor's summary and assessment**	The authors look at current sheets in plasmas (space and laboratory) - these are regions in plasmas that are in between two regions of opposing magnetic field and store magnetic free energy. By means of analytical theory and particle-in-cell simulations, the authors here present insights on what makes these sheets be in equilibrium, or how they relax into equilibrium states.
**Editorial synthesis of reviewer reports**	The 2 referees are generally positive about the work, but have suggestions for improvements. Referee #2 questions the claim of universality, however.

Editorial evaluations

Nature Physics<br><br>Revision not invited<br>Major revisions with extension of the work<br>Major revisions<br>Minor revisions<br>Revisions not needed	The work is of interest to the specialists from the (space) plasma community, but unfortunately, not of sufficiently broad relevance to be suitable for publication in *Nature Physics*.
Nature Communications<br><br>Revision not invited<br>Major revisions with extension of the work<br>Major revisions<br>Minor revisions<br>Revisions not needed	If the authors can make a convincing case that the results are sufficiently universally valid, then *Nature Communications* could be an appropriate venue for publication.
Communications Physics<br><br>Revision not invited<br>Major revisions with extension of the work<br>Major revisions<br>Minor revisions<br>Revisions not needed	If it turns out that the results are not sufficiently universally applicable/valid, then *Communications Physics* would be the best fit.

Next steps

**Editorial recommendation 1:**	Our top recommendation is to revise and resubmit your manuscript to *Nature Communications*, providing the issue of the universality claim can be satisfactorily addressed.
**Editorial recommendation 2:**	You may also choose to revise and resubmit your manuscript to *Communications Physics.* This option might be best if the relevance of the reported work is limited, and no strong case for universality of the results can be made.
**Note**	As stated on the previous page *Nature Physics* is not inviting a revision at this time. Please keep in mind that the journal will not be able to consider any appeals of their decision through Guided Open Access.

Revision

To follow our recommendation, please upload the revised manuscript files using **the link provided in the decision letter**. Should you need assistance with our manuscript tracking system, please contact Adam Lipkin, our Nature Portfolio Guided OA support specialist, at guidedOA@nature.com.

Revision checklist

- ☐ Cover letter, stating to which journal you are submitting
- ☐ Revised manuscript
- ☐ Point-by-point response to reviews
- ☐ Updated Reporting Summary and Editorial Policy Checklist
- ☐ Supplementary materials (if applicable)

Submission elsewhere

If you choose not to follow our recommendations, you can still take the reviewer reports with you.

Option 1: Transfer to another Nature Portfolio journal
Springer Nature provides authors with the ability to transfer a manuscript within the Nature Portfolio, without the author having to upload the manuscript data again. To use this service, **please follow the transfer link provided in the decision letter.** If no link was provided, please contact guidedOA@nature.com.

> *Note that any decision to opt in to In Review at the original journal is not sent to the receiving journal on transfer. You can opt in to In Review at receiving journals that support this service by choosing to modify your manuscript on transfer.*

Option 2: Portable Peer Review option for submission to a journal outside of Nature Portfolio
If you choose to submit your revised manuscript to a journal at another publisher, we can share the reviews with another journal outside of the Nature Portfolio if requested. You will need to request that the receiving journal office contacts us at guidedOA@nature.com. We have included editorial guidance below in the reviewer reports and open research evaluation to aid in revising the manuscript for publication elsewhere.

Annotated reviewer reports

The editors have included some additional comments on specific points raised by the reviewers below, to clarify requirements for publication in the recommended journal(s). However, please note that all points should be addressed in a revision, even if an editor has not specifically commented on them.

**Reviewer #1 information**	
**Expertise**	Space plasmas, magnetic reconnection
**Editor's comments**	The referee is positive about the work reported but has some comments that required addressing in a revised version.

**Reviewer #1 comments**	
**Section**	**Annotated Reviewer Comments**
**Remarks to the Author: Overall significance**	This paper presents a possible process by which to determine the equilibrium outcome of the relaxation of a disequilibrated current sheet in the presence of a small finite guide field. This is an extension of their previous work of the same process without a guide field being present. It is my opinion that this work will provide a basis and framework for future work in current sheet behavior. Although it need not be included in this work, additional study should be conducted with higher current sheet aligned energies and with higher mass species. I feel that this paper should be published after addressing of several minor comments.<br><br>**The referee appreciates the work, is supportive of publication, but lists a series of issues that need to be addressed.**<br><br>General Comment.<br><br>Too many unneeded modifiers. These words obsfucate the message of the paper. This makes it incredibly more difficult to read. Words like “are exhaustively classified” should be “are classified”. “in order to fathom the equilibrium” should be “in order to understand the equilibrium”<br><br>fathom is more than a little ‘over the top’<br><br>Specific Comments. Was this study run in 2D or 3D? I assume 2D since you made “out of plane” streak plots, but this is not explicitly stated.<br><br>FIG 1 is not clear enough to make sense. Consider more traditional line plots of B

and rho as cuts in the YX plane. The arrow widths are nearly impossible to decipher and the pink on pink color choice was just bad. What does it mean when the lines are closer or further apart in d)? Explain this to the reader if you maintain these plots.

In the text body below Figure 1 you explain "Fig 1d exhibits a finite dBz/dx that does not exist in the initial state" How can we see dBz/dx when the only thing shown is B not Bz (which I must assume is total magnetic field strength)? If you do not expect to show dBz/dx in FIG. 1d then you should not refer to it, or refer to the appropriate place.

In FIG. 2, how were the ion orbit trajectories determined? Was this done in 2D or 3D? FIG 2 Do the red (cyan, blue) lines match between column 2 and column 3?

How have you defined meandering?

How does a meandering (M) orbit differ from the other two?

Should there really only be only two kinds of orbits, DW and NC?

Why does meandering in this paper appear to have a different meaning than in your previous paper [29]?

FIG 2 is confusing. Figures a-b-c make sense.

you specified that X is normal to the CS.

Am I correct in assuming that:

1) Z direction is along the CS.

2) Y is across the CS.

3) that your XYZ would be ZXY in GSE coordinates (tail).

As such Column 2 (d-e-f) shows movement along the CS and Column 3 (g-h-i) across the CS? Am I correct in assuming that particle motion is along the CS (by some means) and across the CS due to the Guide Field?

Particles can move in both + and - directions along the current sheet and across the current sheet.

In FIG 2 what decides whether the red, blue or cyan will move: one way or the other (+/-Z) along the CS (d-e-f)?

In FIG 2 what decides whether the red, blue or cyan will move: one way or the other

	(+/-Y) across the CS (g-h-i)?<br><br>I see that later you specify subclasses based on the sign of<br><br>-- Figure 2 The green and magenta colors represent By. I assume the colors are plus and minus but it is not stated. –<br><br>I retract this initial comment and replace it with:<br><br>I suggest that you move the By and Bz arrows above the column or give them a white background. Pink on pink and Green on green is very hard to see.<br><br>What is your XYZ coordinate system? It does not seem to be any standard system such as GSE. Also you are comparing XYZ with LMN which is given in reference to GSE.<br><br>This produces confusion.<br><br>Please clarify how the LMN coordinate system relates to your XYZ system? I mean in general terms like ‘along the CS’, ‘perpendicular to the CS’, ‘across the CS’.<br><br>FIG 4 FIG 5 the color bars could be a little bit wider. They are difficult to see.<br><br>FIG 4 the parentheses ger very confusing when you have embedded ((x,yz)). I understand your desire to try and duplicate statements for two rows that are slightly different.<br><br>Try using e-h {m-p} are respectively the same as a-d, except in (x,vy) {(x,vz)}â€ or similarly [square brackets]. â€œi-l [q-t] Ion current density Jiy [Jiz] obtained by taking the first velocity moment of e-h [mp].â€<br><br>**Taking the above remarks into account when revising the paper should increase its clarity.**
**Remarks to the Author: Impact**	
**Remarks to the Author: Strength of the claims**	
**Remarks to the Author: Reproducibil**	

ity	

Reviewer #2 information	
**Expertise**	Plasma astrophysics, magnetic reconnection
**Editor's comments**	The referee finds the work of importance, interest and impact, but questions the universality of the results as claimed by the authors.

Reviewer #2 comments	
**Section**	**Annotated Reviewer Comments**
**Remarks to the Author: Overall significance**	This paper concerns the problem of relaxation of an out of equilibrium current sheet in collision-less plasmas. The authors are interested in describing how the new equilibrium is selected. They present a theoretical derivation of particle orbits, particle-in-cell numerical simulations, and observations. I don't have any major concerns about the quality of the work or how the paper is currently written.
**Remarks to the Author: Impact**	The subject of the paper is of great importance and potential impact.
**Remarks to the Author: Strength of the claims**	The claim of "universality" that the author makes doesn't seem to be supported enough by the analysis the authors present. In other words, the authors claim that the process they have studied is universal, but the paper does not contain convincing proof of such a statement. For this reason, although the scientific quality of the work is excellent, I don't think that its current version meets the journal requirements.<br><br>**From an editorial point of view, the validity of the universality claim is important in deciding the best journal to publish this work in. If a strong case can be made that there is indeed universality in the results, Nature Communications could be considered as the best venue for the paper. If not, then Communications Physics would be more suitable.**<br><br>In what follows, I list those points that, in my opinion, should be improved to arrive at a more convincing version of this study.<br><br>1. As it is commonly done in PIC simulations, the authors use a nonrealistic speed of light to Alfvén speed ratio (c/va). The results presented in the Extended Data section show that a larger value of c/va results in the production of waves. The author state that these plasma oscillations damp away without affecting the core relaxation mechanisms. I have a few questions on this point. Which kind of waves are these? Why do they develop when a larger c/va is considered? What dumps these waves? What is the

	effect of periodicity on the evolution of these waves inside the system? In an open system, would such waves leave the current sheets? Are these waves observed by MMS or any other satellite?<br><br>2. The simulations the authors have performed are 1D. However, the stability of a single current sheet may depend on 3D dynamics. What justifies the 1D approach the authors use? In their conclusions, the authors also admit that in a 2D case they have studied, reconnection spontaneously develops, which makes questionable the existence in natural environments of a 1D equilibrium like what they are presenting in the paper.<br><br>3. Could the authors better explain why the perpendicular electron temperature in their simulation is everywhere larger than the parallel one (Fig 6 panel e-f) while it is the opposite in the observed current sheet? They make an argument on that just before the section "Discussion and outlook"; however, it was hard for me to understand what they mean.<br><br>4. The authors show that their numerical simulations are in accordance with their analysis of particle trajectories. Would it be possible for them to analyze MMS data and extract the VDF for ions and electrons? How do these compare with those obtained from numerical simulations and the analysis of particle orbits?<br><br>5. At the beginning of page 14, the authors note a discrepancy between the analytical and the numerical solution. This discrepancy seems to be a weak point of the paper since the numerical solution does not relax to one of the expected equilibria. Still, it relaxes to some other equilibrium whose analytical form is unknown. It would be a great result if the authors could find an analytical form corresponding to the final state of their simulations.<br><br>6. In their final discussion, the authors claim that mixed equilibria are likely to be ubiquitous. However, they also admit that their MMS observations concern a current sheet out of equilibrium, and this seems an apparent contradiction. Are the type of equilibria discussed by the authors also frequently observed by MMS?<br><br>**These remarks should help to arrive at an improved version of the apper.**<br><br>Minor comment.<br><br>In Fig 4 and Fig 5 a few distribution functions of the kind f(x,v) are plotted. Could the authors specify how the two not plotted velocities are treated? For instance, when vx is plotted, do they plot a particular plane at fixed vy and vz or the average on vy and vz?
**Remarks to the Author: Reproducibility**	I don't see any significant issue in reproducing the analysis presented in the paper.

Open research evaluation

Guidelines for Transparency and Openness Promotion (TOP) in Journal Policies and Practices ("TOP Guidelines")

The recommendations and requests in the table below are aimed at bringing your manuscript in line with common community standards as exemplified by the TOP Guidelines. While every publisher and journal will implement these guidelines differently, the recommendations below are all consistent with the policies at Nature Portfolio. In most cases, these will align with TOP Guidelines Level 2.

FAIR Principles

The goal of the recommendations in the table below related to **data or code** availability is to promote the FAIR Guiding Principles for scientific data management and stewardship (*Scientific Data* **3**: 160018, 2016). The FAIR Principles are a set of guidelines for improving 4 important aspects of digital research objects: **F**indability, **A**ccessibility, **I**nteroperability and **R**eusability.

ORCID

ORCID is a non-profit organization that provides researchers with a unique digital identifier. These identifiers can be used by editors, funding agencies, publishers, and institutions to reliably identify individuals in the same way that ISBNs and DOIs identify books and articles. Thus the risk of confusing your identity with another researcher with the same name is eliminated. **The ORCID website** provides researchers with a page where your comprehensive research activity can be stored.

Springer Nature collaborates with the ORCID organization to ensure that your research contributions (as authors and peer reviewers) are correctly attributed to you. Learn more at https://www.springernature.com/gp/researchers/orcid

Data availability

Data Availability Statement

Many journals, including all Nature Portfolio journals, require a Data Availability Statement in the manuscript as a condition of publication. The Data Availability Statement should be as detailed as possible and include accession codes or other unique IDs for deposited data, information about where source data can be found, and specify any restrictions to data access that may apply. At a minimum, the statement should indicate that data are available upon request and explain how data access can be granted. If data access is not possible, the reasons for this must be made clear in the Data Availability Statement.

More information about the Nature Portfolio data availability policy can be found here: https://www.nature.com/nature-portfolio/editorial-policies/reporting-standards#availability-of-data

Additional information about Data Availability Statements and Springer Nature's data policies are available here: http://www.springernature.com/gp/authors/research-data-policy/data-availability-statements/12330880

Other data requests

In line with community standards regarding open research, Springer Nature strongly supports data sharing and believes that all datasets on which the conclusions of the paper rely should be available to readers. We encourage authors to ensure that their datasets are either deposited in publicly available repositories (where available and appropriate) or presented in the main manuscript or additional supporting files whenever possible.

To learn more about data sharing and recommended data repositories, please see https://www.springernature.com/gp/authors/research-data-policy/repositories/12327124

Code availability and citation

To adhere to community standards and promote transparency in research, any custom software or code should be made publicly available, ideally before publication so that referees can test the code and comment on it.

Please include a statement under the heading "Code Availability", indicating whether and how the custom code/software reported in your study can be accessed, including any restrictions to access. This section should also include information on the versions of any software used, if relevant, and any specific variables or parameters used to generate, test, or process the current dataset. Code availability statements should be provided as a separate section after the Data Availability section.

Upon publication, Nature Portfolio journals consider it best practice to release custom computer code in a way that allows readers to repeat the published results. Code should be deposited in a DOI-minting repository such as Zenodo, Gigantum or Code Ocean and cited in the reference list following the guidelines described in our policy pages (see link below). Authors are encouraged to manage subsequent code versions and to use a license approved by the open source initiative. Full details about how the code can be accessed and any restrictions must be described in the Code Availability statement.

See here for more information about Nature Portfolio's code availability policies:
https://www.nature.com/nature-portfolio/editorial-policies/reporting-standards#availability-of-computer-code

We also provide a Code and Software submission checklist that you may find useful:
https://www.nature.com/documents/nr-software-policy.pdf

Please note: because of advanced features used in this form, you must use Adobe Reader to open the document and complete it.
